# Milrinone in congenital diaphragmatic hernia – a randomized pilot trial: study protocol, review of literature and survey of current practices

**DOI:** 10.1186/s40748-017-0066-9

**Published:** 2017-11-27

**Authors:** Satyan Lakshminrusimha, Martin Keszler, Haresh Kirpalani, Krisa Van Meurs, Patricia Chess, Namasivayam Ambalavanan, Bradley Yoder, Maria V. Fraga, Holly Hedrick, Kevin P. Lally, Leif Nelin, Michael Cotten, Jonathan Klein, Stephanie Guilford, Ashley Williams, Aasma Chaudhary, Marie Gantz, Jenna Gabrio, Dhuly Chowdhury, Kristin Zaterka-Baxter, Abhik Das, Rosemary D. Higgins

**Affiliations:** 1 University at Buffalo, Buffalo, NY USA; 2 Women and Infants Hospital, Providence, RI USA; 3 Children’s Hospital of Philadelphia, Philadelphia, PA USA; 4 Stanford University, Palo Alto, CA USA; 5 University of Rochester, Rochester, NY USA; 6 University of Alabama at Birmingham, Birmingham, AL USA; 7 University of Utah, Salt Lake City, UT USA; 8 University at Texas at Houston, Houston, TX USA; 9 Nationwide Children’s Hospital, Columbus, OH USA; 10 Duke University, Durham, NC USA; 11 University of Iowa, Iowa City, IA USA; 12 RTI, Research Triangle Park, NC USA; 13 NICHD, Neonatal Research Network, Bethesda, MD USA

**Keywords:** Oxygen, Phosphodiesterase, Pulmonary hypertension, Persistent pulmonary hypertension, Extracorporeal membrane oxygenation

## Abstract

**Background:**

Congenital diaphragmatic hernia (CDH) is commonly associated with pulmonary hypoplasia and pulmonary hypertension (PH). PH associated with CDH (CDH-PH) is frequently resistant to conventional pulmonary vasodilator therapy including inhaled nitric oxide (iNO) possibly due to right and left ventricular dysfunction. Milrinone is an intravenous inotrope and lusitrope with pulmonary vasodilator properties and has been shown anecdotally to improve oxygenation in PH. We developed this pilot study to determine if milrinone infusion would improve oxygenation in neonates ≥36 weeks postmenstrual age (PMA) with CDH.

**Methods/design:**

Data on pulmonary vasodilator management and outcome of CDH patients was collected from 18 university NICUs affiliated with the Neonatal Research Network (NRN) from 2011 to 2012. The proposed pilot will be a masked, placebo–controlled, multicenter, randomized trial of 66 infants with CDH with an oxygenation index (OI) ≥10 or oxygen saturation index (OSI) ≥5. The primary outcome is the oxygenation response, as determined by change in OI at 24 h after initiation of study drug. As secondary outcomes, we will determine oxygenation at 48 h and 72 h post-infusion, right ventricular pressures on echocardiogram and the incidence of systemic hypotension, arrhythmias, intracranial hemorrhage, survival without extracorporeal membrane oxygenation, and chronic lung disease (oxygen need at 28 days postnatal age). Finally, we will evaluate the pulmonary and nutritional status at 4, 8 and 12 months of age using a phone questionnaire.

**Results:**

Three hundred thirty-seven infants with CDH were admitted to NRN NICUs in 2011 and 2012 of which 275 were ≥36 weeks PMA and were exposed to the following pulmonary vasodilators: iNO (39%), sildenafil (17%), milrinone (17%), inhaled epoprostenol (6%), intravenous epoprostenol (3%), and intravenous PGE1 (1%). ECMO was required in 36% of patients. Survival to discharge was 71%.

**Discussion:**

CDH is an orphan disease with high mortality with few randomized trials evaluating postnatal management. Intravenous milrinone is a commonly used medication in neonatal/pediatric intensive care units and is currently used in 17% of patients with CDH within the NRN. This pilot study will provide data and enable further studies evaluating pulmonary vasodilator therapy in CDH.

**Trial registration:**

ClinicalTrials.gov; NCT02951130; registered 14 October 2016.

## Background

Congenital diaphragmatic hernia (CDH) is one of the common serious congenital anomalies managed in the neonatal intensive care unit (NICU) [1]. Approximately 1600 newborn infants with CDH are born in the United States every year (http://www.cherubs-cdh.org/), and CDH is an orphan disease that is excluded from many trials involving persistent pulmonary hypertension of the newborn (PPHN). Infants with CDH usually have pulmonary hypoplasia (underdeveloped lungs) and pulmonary hypertension (CDH-PH) leading to hypoxemic respiratory failure (HRF). The presence of other anomalies (especially cardiac defects), location of the liver (intraabdominal vs. herniation in part to the thorax), degree of pulmonary hypoplasia and side of the hernia defect (right or left sided) may be associated with a different prognosis [2].

**Fig. 1:**
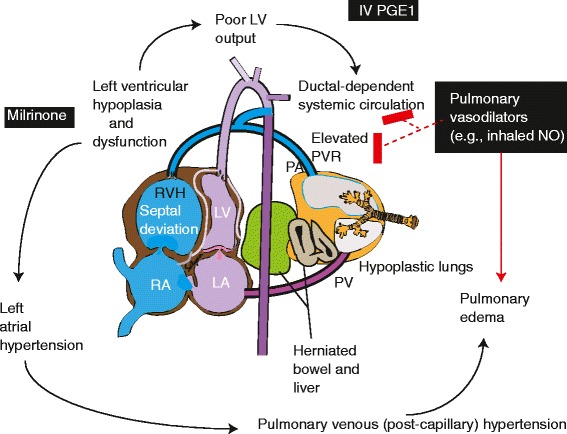
Patients with left sided CDH may have left ventricular hypoplasia and dysfunction. Such dysfunction may be associated with elevated left atrial pressure, pulmonary venous hypertension and poor LV output. The systemic circulation may be dependent on right to left ductal flow due to elevated PVR. Pulmonary vasodilators such as inhaled NO may result in pulmonary arterial dilation and exacerbate pulmonary edema in the presence of pulmonary venous hypertension and decrease ductal-dependent systemic flow (“ductal steal”). IV PGE1 maintains ductal patency leading to reduced RV afterload and support systemic circulation. Milrinone, by improving left ventricular diastolic and systolic function reduces left atrial pressure and also dilates pulmonary vasculature resulting in improved oxygenation in CDH. The presence of hypoplastic lungs with remodeled pulmonary vasculature and volutrauma, barotrauma and oxygen toxicity contribute to poor response to pulmonary vasodilator therapy. We hypothesize that a combination of milrinone with “gentle” ventilation will improve oxygenation and response to pulmonary vasodilator therapy in CDH. RVH – right ventricular hypertrophy; RA – right atrium; LA – left atrium; LV – left ventricle: PVR – pulmonary vascular resistance; NO – nitric oxide; PA – pulmonary artery; PV – pulmonary vein; IV PGE1 – intravenous prostaglandin E1 (alprostadil)

Despite many technological advances over the past two decades, including high frequency ventilation, inhaled nitric oxide (iNO) and extracorporeal membrane oxygenation (ECMO), CDH presenting in the neonatal period continues to be associated with relatively high rates of morbidity and mortality (28–30%) [1, 3]. Survival of infants at tertiary care centers automatically selects for patients who survive birth, resuscitation, and transport from an outside facility, and therefore likely underestimates the true hidden mortality associated with CDH. The major underlying pathophysiology in such infants appears to be a combination of lung immaturity, hypoplasia, and CDH-PH, which may be further aggravated by left ventricular underdevelopment [4–7] (Fig. 1).

**Fig. 2:**
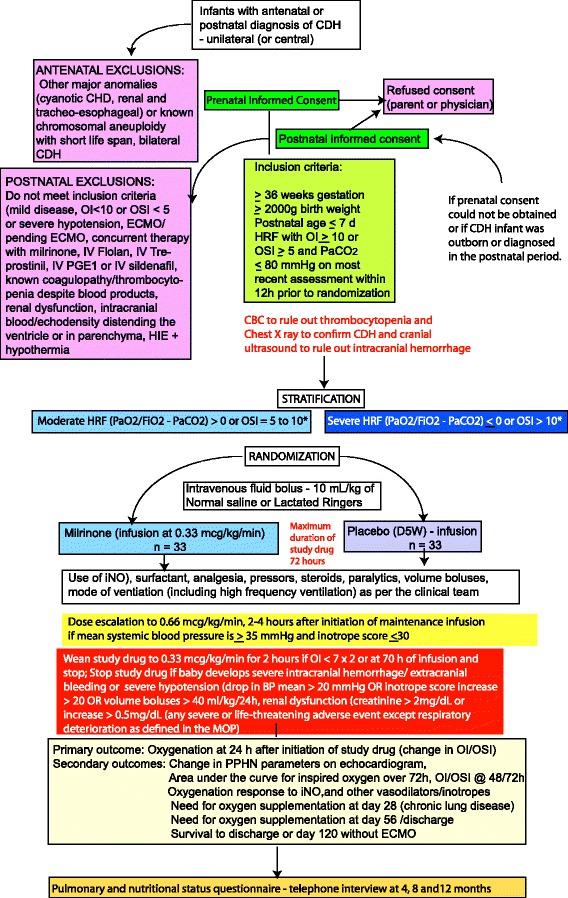
Study design – please see text for details. * In the presence of an indwelling arterial catheter, or if there is an arterial blood gas drawn by arterial stick, oxygenation index (OI) values are preferred for making decisions regarding study drug therapy over oxygen saturation index (OSI) values

**Fig. 3:**
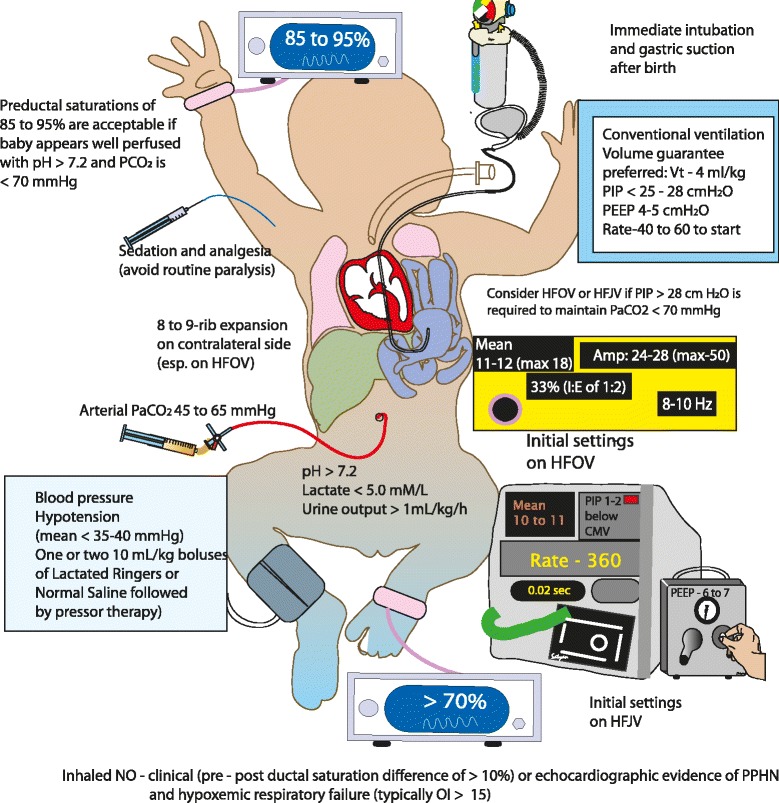
Optional guidelines for “optimal” management of a neonate with congenital diaphragmatic hernia in the preoperative period. A nasogastric or orogastric suction tube is placed to decompress the stomach in the delivery room. Respiratory management includes intubation and positive pressure ventilation with care to avoid high PIP. The target of respiratory management is to maintain preductal oxygen saturations in the 85–95% range and PaCO_2_ between 45 and 70 mmHg with a pH > 7.20. If PaCO_2_ of ≤70 mmHg cannot be achieved with conventional ventilation (maximum PIP of 28 cm H_2_O and a maximum rate of 60/min), high frequency ventilation (high frequency oscillator – HFOV or jet ventilator – HFJV) may be required. Blood pressure is maintained to achieve adequate perfusion and avoid lactic acidosis and oliguria. Monitoring chest X-rays to maintain contralateral lung expansion to 8 to 9 ribs may avoid baro/volutrama. A trial of inhaled NO may be considered when oxygenation index exceeds 15 with clinical or echocardiographic evidence of pulmonary hypertension. CMV – conventional mechanical ventilation. Modified from Chandrasekharan et al. [48]

**Fig. 4:**
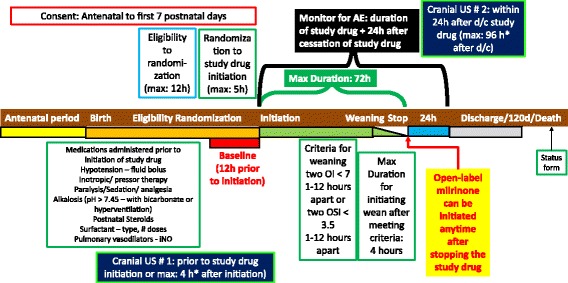
Protocol for the milrinone CDH study. Parents are approached for consent during either the antenatal period or the first 7 postnatal days. Once eligibility is determined based on presence of hypoxemic respiratory failure and meeting all the inclusion criteria and absence of any of the exclusion criteria, subjects are randomized to milrinone or placebo. Details of other modalities of treatment (alkalosis, surfactant), medications administered (including pulmonary vasodilator therapy, vasopressor therapy and postnatal steroids) are recorded. A baseline cranial ultrasound (#1) is obtained either prior to or within 4 h of study drug initiation. Randomization is performed within 12 h of establishing eligibility. The study drug should be initiated within 5 h of randomization. Maximum duration of study drug therapy is 72 h. Patient is monitored for adverse events (AE) 24 h after cessation of study drug. The criteria for triggering study drug wean/discontinuation are two oxygenation indices (OI) < 7 at least an hour or maximum 12 h apart. If arterial access is not available, oxygen saturation index (OSI) will be used to assess hypoxemia. Once weaning/discontinuation criteria are met, study drug should be weaned within 4 h. Open label milrinone can be initiated any time after completion of the study drug. Status form (including data on surgical details) will be collected at the end of the hospital course or at 120 days of postnatal age or death (whichever is earlier)

**Fig. 5:**
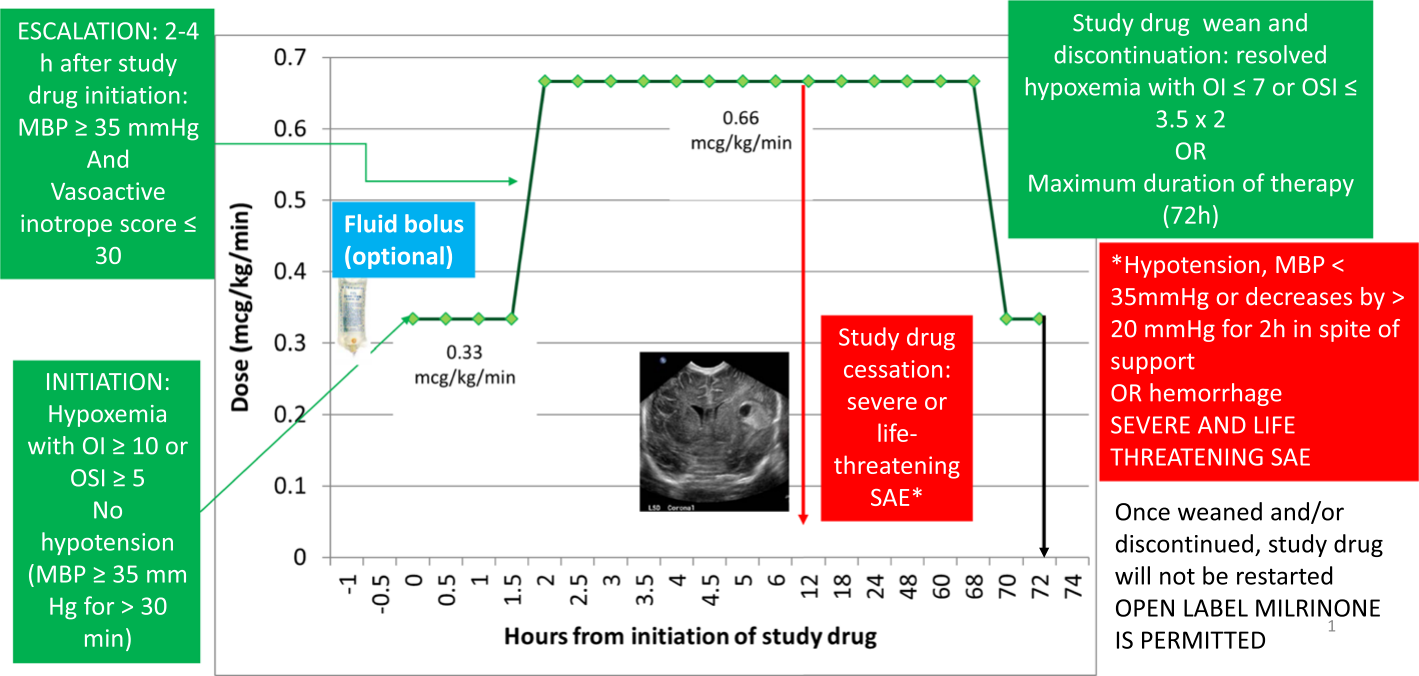
Protocol for initiation, escalation, weaning and discontinuation of study drug. The study drug (milrinone or placebo – D5W) is initiated after documentation of hypoxemia (oxygenation index – OI ≥ 10 or oxygen saturation index – OSI ≥ 5), in the absence of hypotension and other exclusion criteria. A fluid bolus (10 ml/kg of lactated Ringers solution or normal saline) is recommended prior to study drug initiation. The starting dose is 0.33 μg/kg/min. After 2–4 h of therapy at this dose, study drug is escalated to 0.66 μg/kg/min in the absence of hypotension (mean systemic BP ≥ 35 mmHg and vasoactive inotrope score ≤ 30). The maximum duration of therapy is 72 h. Study drug is weaned to 0.33 μg/kg/min when study discontinuation criteria are met (two OIs < 7 or two OSIs <3.5 at least an hour apart). After 2 h of study drug infusion at 0.33 μg/kg/min, study drug can be discontinued. The presence of any severe and life threatening serious adverse events (SAE) necessitates immediate cessation of study drug. Open label milrinone is permitted after cessation of study drug

### Randomized trials in preoperative management of CDH

In spite of such high mortality, a randomized controlled trial targeting iNO use in CDH-PH [the Inhaled nitric oxide and hypoxic respiratory failure in infants with congenital diaphragmatic hernia (NINOS)] [8] and one trial targeting mode of ventilation have been published to date (VICI trial) [9, 10]. The NINOS-CDH study was conducted through the Neonatal Research Network (NRN) and the Canadian Institute of Health Research over 15 years ago. In the past decade following the completion of the NINOS-CDH study, significant changes in mechanical ventilation, such as a ‘gentle’ approach to ventilation of newborn infants, recognition of oxygen toxicity, tolerance to lower SpO_2_ and higher PaCO_2_ (permissive hypercapnia) and delayed surgical repair have changed the management of CDH. The NINOS-CDH study failed to demonstrate any benefit of iNO; in fact, the need for ECMO was significantly increased in iNO treated infants. Data from the extracorporeal life support organization (ELSO) registry (http://www.elsonet.org/) indicate that CDH, in addition to being the most common indication for neonatal respiratory ECMO (29.8% of cases), is also the diagnosis associated with the highest mortality (50.5% between July 2008 and June 2013) in infants who had undergone ECMO for neonatal respiratory indications. Regrettably, in patients with CDH on ECMO, survival has decreased from 53.4% during 1990–2000 to 45.4% (*p* < 0.0001) during 2001 to 2011 [11]. Survivors with CDH have long-term problems including pulmonary hypertension, reflux disease [12, 13] and chronic lung disease. [14]

A second trial randomized infants with prenatally diagnosed CDH into high frequency oscillation (*n* = 80) versus conventional mechanical ventilation (*n* = 91) as the initial mode of ventilation in Europe [10]. There was no difference in primary outcome of mortality/oxygen need at 28 days postnatal period (referred to as chronic lung disease in the current manuscript). However, patients on conventional ventilation as the initial mode had less use of ECMO, iNO, and sildenafil. The duration of vasoactive drugs was shorter in patients randomized to conventional ventilation. A third trial evaluating the use of sildenafil in chronic pulmonary hypertension associated with CDH was recently terminated at University of California, San Francisco (NCT00133679). The change in clinical practice allowing chronic therapy with sildenafil, incompatible with possibility of placebo is listed as the reason for termination of this study. A trial evaluating IV Treprostinil (Remodulin, NCT02261883) and one evaluating IV sildenafil (NCT01720524) in PPHN are recruiting and include patients with CDH-PH. To our knowledge, there are no other trials currently evaluating milrinone therapy in CDH-PH.

Milrinone is a phosphodiesterase 3 (PDE3) inhibitor and is currently approved for adult patients with heart failure. In addition to its inotropic and lusitropic effects on the heart, it is also a pulmonary vasodilator [15, 16]. It causes systemic vasodilation and reduces afterload in patients with heart failure. Vascular PDE3 breaks down cyclic adenosine monophosphate (cAMP) in arterial smooth muscle cells and the myocardium. By inhibiting PDE3, milrinone increases cAMP levels in cardiac muscle and vascular cells; it functions as a vasodilator and improves ventricular function directly and by decreasing afterload. Animal models of PPHN demonstrate increased PDE3 expression, which could promote vascular dysfunction [17]. Recent laboratory studies also indicate that hyperoxic ventilation with iNO dramatically increases PDE3 activity [18], suggesting that these therapies may reduce cAMP and paradoxically contribute to vascular dysfunction [19]. Such enhanced PDE 3 levels, in theory, augment the effectiveness of milrinone as a pulmonary vasodilator. Milrinone inhibits PDE3 and enhances cAMP levels and promotes vasodilation of pulmonary arterial rings in animal models of PPHN [20].

### Studies evaluating milrinone use in children

The benefits of milrinone therapy in children following surgery for congenital heart disease have been well established in several studies (including the randomized, double-blind Prophylactic intravenous use of milrinone after cardiac operation in pediatrics (PRIMACORP) study, with 238 infants) [21–24]. In the PRIMACORP study among 238 treated patients, 25.9%, 17.5%, and 11.7% in the placebo, low-dose milrinone, and high-dose milrinone groups, respectively, developed low cardiac output syndrome in the first 36 h after cardiac surgery. High-dose milrinone significantly reduced the risk of development of low cardiac output syndrome compared with placebo, with a relative risk reduction of 55% (*P* = 0.023) in 238 treated patients and 64% (*P* = 0.007) in 227 patients without major protocol violations.

### Milrinone in PPHN

Anecdotal reports have shown that milrinone can be an effective therapeutic option in PPHN [25, 26]. Three retrospective case reports from two different hospitals in Ontario, Canada evaluated late preterm/term infants [except one infant at 26 weeks postmenstrual age (PMA)] with HRF or PPHN unresponsive to iNO. Bassler et al. reported four infants with PPHN. Some of the infants were “primed” with normal saline (15 ml/kg) and administered a loading dose of milrinone at 50 μg/kg over 30 min followed by a 0.33 μg/kg/min infusion. None of the infants developed systemic hypotension, and all of them showed consistent improvement in oxygenation. One of the infants was born at 26 weeks PMA and developed bilateral intraventricular hemorrhage (IVH) with moderate dilation of all ventricles. Another term infant developed an IVH. The third infant (39 weeks PMA) showed a small left subependymal hemorrhage. [27] McNamara et al. reported 9 term infants with PPHN and poor response to iNO who received IV milrinone. [25] Because of the potential risk of systemic hypotension, a loading dose was avoided in these patients with PPHN. The infusion was started at 0.33 μg/kg/min and increased in increments of 0.33 μg/kg/min according to clinical response to a maximum of 0.99 μg/kg/min. There was a significant improvement in oxygenation after commencement of milrinone, particularly in the first 24 h of infusion. Tachycardia improved and there was no systemic hypotension. More recently, the same authors performed pharmacokinetic studies in 11 late preterm and term infants with PPHN resistant to iNO with a loading dose of 50 μg/kg over 60 min followed by an infusion of 0.33 to 0.99 μg/kg/min and demonstrated an improvement in oxygenation and cardiac output by echocardiography, as well as a reduction in pulmonary arterial pressure without any associated IVH [28]. Milrinone improves right ventricular function and reduces strain and increases right and left ventricular output in infants with PPHN. [29] The safety and pharmacokinetics of milrinone in preterm infants has been evaluated [30–32]. The available information on the pharmacokinetics of milrinone in different age groups is presented in Table 1.

**Table 1:** Pharmacokinetic data on milrinone in various age groups - modified from Lakshminrusimha et al. [43]

Age group	Loading dose	Maintenance dose	Half-life (h)	Total body clearance (mL/kg/min)	Volume of distribution (L/kg)
Adult [44]	12.5 to 75 μg/kg	0.5 μg/kg/min	0.8 ± 0.22	6.1 ± 1.3	0.32 ± 0.08
Child [45]	25 to 75 μg/kg	0.25 to 0.75 μg/kg/min	3.7	2.5 to 10.6 (increases with age)	0.7–0.9
Neonate (post-op CHD) [21]	25 μg /kg over 60 min (low dose) 75 μg /kg (high dose)	0.25 μg /kg/ min (low dose) 0.75 μg /kg/min (high dose)		1.64 ± 0.37	0.523 ± 0.028
Neonate (PPHN) [46] [47]	50 μg /kg over 60 min	0.33 (to 0.99) μg /kg/min	4.1 ± 1.1	1.83 ± 0.17 to 3.05	0.56 ± 0.19
Preterm neonate [32]	0.75 μg /kg/min for 3 h	0.2 μg /kg/min	10.3	0.64	0.576

### Rationale for use of milrinone in CDH

The poor oxygenation response to iNO and other vasodilators in infants with CDH may be due to a combination of right and left ventricular dysfunction. Remodeled pulmonary vasculature in CDH results in severe PH that leads to right ventricular dysfunction. An inotropic vasodilator such as milrinone improves right ventricular diastolic function and increases right ventricle systolic velocity in CDH resulting in improved pulmonary blood flow and oxygenation [33]. Recently, a case series from Australia suggested that right ventricular diastolic dysfunction measured by tissue Doppler on day 1 and 2 of life correlates with length of stay and duration of respiratory support [34] in infants with CDH. Compared to other causes of PPHN, infants with left sided CDH have significantly lower left ventricular mass; and it has been noted that infants who required ECMO had a lower left ventricular mass than those who did not require ECMO [7, 35]. We speculate that the reduced left ventricle mass is related to dysfunction, resulting in increased left atrial pressure and pulmonary venous hypertension. This may explain why iNO and other pulmonary arterial dilators are less effective in infants with CDH. [36] These agents can often worsen oxygenation in the presence of pulmonary venous hypertension and/or cardiac dysfunction [37]. Milrinone, an “inodilator” (inotrope with vasodilator properties) [16], may improve left ventricular function and potentially promote pulmonary vasodilation in infants with CDH (Fig. 1).

## Methods/design (Fig. 2)

This pilot study is designed as a prospective, randomized, placebo-controlled, multicenter trial with a 1:1 randomization to milrinone or placebo (5% dextrose infusion). All participating centers are part of the Neonatal Research Network (NRN). The NRN is a well-established Eunice Kennedy Shriver National Institute of Child Health and Human Development (NICHD) funded network of 15 universities and affiliated academic hospitals across the US that has collaborated to improve neonatal care.

### Outcome measures

The primary outcome is the oxygenation response, as determined by change in oxygenation index (OI = mean airway pressure in cmH_2_O X FiO_2_ X 100 ÷ PaO_2_) at 24 h after initiation of study drug. In patients that require ECMO or die prior to completion of 24 h from the initiation of the study drug, the last OI prior to initiation of ECMO or death will be used for analysis. In subjects without an indwelling arterial line, or access to an arterial gas by an arterial stick, oxygen saturation index (OSI = mean airway pressure in cm H_2_O X FiO_2_ X 100 ÷ preductal systemic oxygen saturation - SpO_2_) will be calculated using representative SpO_2_ values recorded at protocol-specified time points. In subjects with an arterial line, OSI will be calculated at the time of the ABG sampling. In subjects without an arterial line, the stable pulse oximeter reading (both preductal and postductal, if available) closest to the beginning of the 6 h window will be recorded. If the study drug was started at 9 am and the 6–12 h window is from 3 pm to 9 pm, the preductal SpO2 close to 3 pm will be used for this period.

Secondary outcomes include:

(i) Oxygenation response at 24 h after initiation of study drug assigning a minimum OI of 40 or OSI of 20 for subjects that need ECMO or die after initiation of study drug infusion but prior to 24 h of study-drug infusion. In this analysis, if the patient’s OI is 30 (or OSI of 15) and the patient is cannulated for ECMO due to hemodynamic instability, he/she will be assigned an OI of 40 (or OSI of 20) for calculating this outcome. However, if the OI prior to ECMO is 47 (i.e., > 40), the higher number (47) will be used for analysis of this secondary outcome. This secondary outcome was added as some infants with CDH are placed on ECMO for hemodynamic instability at a lower OI/OSI.
(ii) Oxygenation index (or OSI) at 48 and 72 h (or OI at the time of initiation of ECMO or death, for infants placed on ECMO or who die before these time points)
(iii) Area under the curve for inspired oxygen after initiation of the study drug (inspired oxygen and ventilator data from 4 time points per day – every 6 h will be recorded to calculate area under the curve)
(iv) Change in echocardiographic findings – velocity of tricuspid regurgitation, left ventricular ejection fraction, position of the interventricular septum and direction of shunt at the PDA and interatrial shunt – prior to and 24–72 h after starting the study medication
(v) Vasoactive Inotrope score (defined as a quantitative assessment of the degree of therapeutic support required by the patient to maintain adequate perfusion and/or blood pressure. The score is calculated as follows: (dose of dopamine in μg/kg/min + dose of dobutamine in μg/kg/min + 100 X epinephrine dose in μg/kg/min + 100 X norepinephrine dose in μg/kg/min + 100 X phenylephrine dose in μg/kg/min + vasopressin dose in U/kg/min × 10,000). We will also evaluate systemic blood pressure changes with the study drug.
(vi) If subsequent to initiation of the study drug, additional inotropes were used (such as dopamine, dobutamine, vasopressin, epinephrine and norepinephrine), an inotrope score will be calculated daily for 3 days from initiation of study drug [38]
(vii) If additional pulmonary vasodilator therapy is used (such as iNO), we will evaluate the oxygenation response to these agents. The OI and PaO_2_/FiO_2_ ratio prior to and after initiation of the inotrope/vasodilator will be recorded. The change in OI and PaO_2_/FiO_2_ ratio in response to these agents will be evaluated as a continuous variable and arbitrarily classified into responders, partial responders and non-responders similar to prior trials [39, 40]
  - complete response is defined as an increase in PaO_2_/FiO_2_ ratio > 20 mmHg from baseline
  - partial response is an increase in PaO_2_/FiO_2_ ratio of 10 to 20 mmHg from baseline
  - no response is defined as an increase of <10 mmHg (or a decrease) in PaO_2_/FiO_2_ ratio from baseline
  - the site of sampling (preductal or postductal) will be recorded
  - in subjects without arterial access, an OSI will be used to determine response to vasodilators
(viii) Supplemental oxygen at 28 days, and 56 days and at discharge (or day 120, whichever time point is earlier).
(ix) Survival to discharge (or day 120, whichever comes earlier) without ECMO.
(x) Clinical status – pulmonary (use of supplemental oxygen or respiratory medications – pulmonary vasodilators), diuretics, methylxanthines, steroids, inhaled or nebulized steroids or bronchodilators, and nutritional (weight, length, head circumference, use of anti-reflux medications) at 4, 8, and 12 months of age.
(xi) Feasibility to perform definitive trial with the primary outcome of improved survival without ECMO. The following factors will be considered: slow recruitment, length of time for recruitment, number of serious adverse events reported, and interest from non-NRN hospitals or international collaboration in performing a definitive trial.

### Inclusion criteria

Infants are eligible if they meet *all* of the following criteria:

- Antenatal or postnatal diagnosis of CDH confirmed with a chest x-ray
- ≥ 36 0/7 weeks postmenstrual age (PMA) by best obstetric estimate and birth weight ≥ 2000 g
- Postnatal age ≤ 7 days
- Invasive mechanical ventilation for HRF secondary to CDH with OI ≥ 10 (or OSI ≥ 5) on the most recent arterial blood gas (ABG) within 12 h prior to randomization
  - An acute reversible mechanical factor such as endotracheal tube obstruction or pneumothorax as a cause of elevated OI or OSI should be ruled out prior to randomization
- At least one blood gas with PCO_2_ ≤ 80 mmHg on the most recent blood gas within 12 h prior to randomization

### Exclusion criteria

Infants are ineligible if they meet *any* of the following criteria:

- Antenatal or postnatal diagnosis of bilateral CDH
- Known cyanotic congenital heart disease (acyanotic conditions such as patent ductus arteriosus - PDA, atrial septal defect - ASD and ventricular septal defect – VSD are included)
- Hypertrophic cardiomyopathy
- Enrolled in conflicting clinical trials (mothers enrolled in fetal tracheal occlusion studies such as FETO may be enrolled if permitted by investigators of the fetal tracheal occlusion study)
- Associated abnormalities of the trachea or esophagus (e.g., trachea-esophageal fistula, esophageal atresia, laryngeal web, tracheal agenesis)
- Renal anomalies resulting in renal dysfunction or severe oligohydramnios (Amniotic Fluid Index <5) associated with renal dysfunction (with serum creatinine >2 mg/dL, not due to maternal factors)
- Decision is made to provide comfort/palliative care and not full treatment
- Known large intraventricular hemorrhage resulting in distension of the ventricle or intraparenchymal bleed or any intracranial hemorrhage associated with a midline shift
- Thrombocytopenia (platelet count <80,000/mm^3^) or coagulopathy (INR > 1.7) despite blood product administration on the most recent blood draw
- Hemodynamic instability with severe systemic hypotension (mean blood pressure < 35 mmHg for at least 2 h with a vasoactive inotrope score of >30
- Aneuploidy associated with short life span (such as trisomy 13 or 18)
- Use of milrinone infusion prior to randomization
- Infants on intravenous pulmonary vasodilator therapy (such as sildenafil or prostacyclin analogs including PGE1, epoprostenol, or treprostinil) prior to initiation of study drug
- Therapeutic hypothermia for hypoxic-ischemic encephalopathy
- Subjects already on ECMO or patients who are being actively considered for ECMO
- Attending (neonatal, critical care or surgical) refusal for participation in the trial (including concern about presence of hemodynamic instability)

### Statistical analysis

#### Sample size

In the case series describing the effect of milrinone among neonates with CDH, baseline OI (mean ± SD) was 10.6 ± 5.6, and improved to 7.9 ± 6.2 and 5.1 ± 2.6 at 12–24 h and at 48–72 h, respectively [33]. Assuming that (a) children receiving placebo will have no additional change in OI (mean change in OI = 0 at 24 h), (b) that children receiving milrinone will improve to a mean OI of 6.0 (a mean change in OI of 4.6) at 24 h, and (c) that the standard deviation of the change from baseline OI at 24 h in each group is 6.2, 33 subjects in the control group and 33 subjects in the milrinone group would have 84% power to detect this difference with a two-sided alpha of 0.05. Phrased differently, if both the control and treatment OI’s were to change, for any given difference in OI, the sample size would be sufficient to detect an effect size of 0.7 with a power of 84%. The sample size will be sufficient to provide estimates of adverse event rates in the treatment group.

#### Stratification based on assessment of severity of CDH

There is wide variation in CDH severity and asymmetric distribution of severity between the control arm and a treatment arm of a randomized trial needs to be avoided. Patients will be stratified based on the severity of HRF into two categories at the time of randomization.

- Moderate HRF: PaO_2_/FiO_2_ ratio – PaCO_2_ value ≥0.
- Severe HRF: PaO_2_/FiO_2_ ratio – PaCO_2_ value <0 [41]. The overall mortality for CDH in a study evaluating PaO_2_ – PaCO_2_ as a predictor of mortality was 30.7%. If the initial (PaO_2_ – PaCO_2_) value was <0, mortality was 55.3%. [41]
- In subjects without an ABG, moderate HRF is defined as a preductal OSI of 5 to 10 and severe HRF is defined as an OSI > 10.

#### Details of the planned analysis

Baseline data will be collected prior to randomization including gestational age, side of the diaphragmatic hernia, birth weight, mode of delivery and arterial blood gas parameters (oxygenation index-OI) and/or pulse oximetry data (oxygen saturation index-OSI). Data from a baseline clinical echocardiogram is recorded (if performed prior to study drug initiation or first echocardiogram performed after initiation of study drug). During the infusion of study drug, arterial blood gas parameters and hemodynamic data (blood pressure, heart rate and echocardiography results) will be recorded. After completing the study infusion, surgical details, and mortality information will be collected. Among survivors, respiratory and nutritional status at discharge and at 4, 8 and 12 months of age will be collected.

Data will be presented as mean ± SD for normally distributed variables and as median and ranges (with percentiles) for non-normally distributed data. All analysis will be by intention-to-treat. Baseline characteristics will be compared between treatment groups using chi-square and Fisher’s exact test for discrete variables and t-test and ANOVA for continuous variables. The primary outcome, difference from baseline OI at 24 h, will be compared between treatment groups using a general linear model adjusted for the stratification factor of HRF severity. Other continuous outcomes will be analyzed similarly. Models for longitudinal outcomes such as oxygenation and vasoactive inotrope scores (short-term) and nutritional and pulmonary outcomes at 12 months (long-term) will include the effect of time, the interaction between time and treatment, and random effects to account for the correlation between measures taken on the same individual over time. Categorical outcomes will be analyzed using analogous generalized linear models. Cox regression models (with adjustment for HRF severity) will be used to plot proportion of responders who survive without ECMO against survival time in hours. Analyses may adjust for clinical site if treatment assignments are severely imbalanced for some sites provided that it is computationally feasible to do so. The potential influence of baseline measures such as time of diagnosis – antenatal vs. postnatal, side of the defect, and gender on pulmonary and nutritional outcome will be explored through the use of additional models similar to those described above.

#### Sample size limitations

This pilot study is not powered to compare rates of the outcome of death or ECMO between groups. To detect a reduction of 10% (from 40% incidence of death/ECMO in control arm to 30% incidence in the milrinone arm), with a 1:1 ratio of treatment and control subjects, a power of 0.8 and an alpha value of 0.05, we would require 376 subjects in each arm. We intend to obtain preliminary data for a definitive trial with a primary outcome of death/ECMO. The study is not powered to compare a sicker cohort of CDH (OI ~ 40s) patients similar to that evaluated in the CDH NINOS study. The patients in this study had an OI of approximately 45 with standard deviations of 16.3 and 14.5 in control and treatment groups respectively. Such a cohort will require a sample size of 207 patients in each arm.

#### Arrangements for randomization, allocation concealment and blinding

Central randomization with computer random number generation will be implemented with 1:1 allocation, stratified by severity of HRF. Stratification based on center and side of the defect is ideal. Since this is a relatively small trial, stratification is not possible for these two variables. The allocation will be unknown to the members of the clinical and research teams and all staff at the coordinating center except the pharmacist compounding the medication.

### Study procedures

Management of infants with CDH in the study is based on individual center protocols or as per individual physician/provider preference. Suggested guidelines for management are shown in Fig. 3. Mothers with an antenatal diagnosis of fetal CDH and infants admitted to participating NICUs with a postnatal diagnosis of CDH will be approached for screening and consent (Fig. 4). In many institutions, mothers are referred by the Obstetrician and/or Maternal-Fetal Medicine (MFM) specialist for a consult with Pediatric Surgery and Neonatology. This would be an ideal time to obtain consent. Mothers admitted to labor and delivery with an antenatal diagnosis may be approached for consent (if not in active labor or on narcotic analgesia). Infants that are diagnosed postnatally, or born to mothers that could not be consented during the prenatal period or transferred from outside hospitals may be approached in the first 168 h of postnatal life (7 days). Using inclusion and exclusion criterion, study staff will determine if the infant is eligible for randomization. Eligible infants will be stratified based on the severity of HRF into the moderate or severe categories at the time of randomization. Infants will be randomized to either the milrinone arm or the placebo arm using a web-based system in a 1:1 ratio. Once the randomization code is received, the study pharmacist at the center will prepare an infusion of study drug. Infusion is initiated at 0.33 μg/kg/min (19.8 μg/kg/h; about 0.1 ml/kg/h using the premixed bag 200 μg/ml milrinone in 5% dextrose solution) after priming the infusion circuit. An equivalent volume of 5% dextrose will be used for infants randomized to the placebo arm. The premix solution in 5% dextrose has a pH of 3.2 to 4.0.

#### Dose

Milrinone/placebo will be initiated as an infusion of 0.33 μg/kg/min (Fig. 5). It is recommended that infants receive a fluid bolus (Lactated Ringers solution or 0.9% normal saline 10 mL/kg) *before* initiating study drug to avoid systemic hypotension, unless there is a contraindication. However, this is optional and based on clinical team decision. If after 2–4 h of infusion at this dose, no evidence of hypotension (defined as mean blood pressure ≥ 35 mmHg and a vasoactive inotrope score of ≤30) is observed, the rate of the infusion is increased to 0.66 μg/kg/min. Invasive blood pressure measurements through an indwelling arterial line (if available) are preferred to non-invasive assessment of blood pressure.

#### Compatibility

Milrinone is compatible with 5% dextrose, normal saline and Lactated Ringers solution. Milrinone is incompatible with chlorothiazide, furosemide, imipenem/cilastatin and procainamide. Detailed compatibility information is listed in Table 2.

**Table 2:** Terminal site compatibility with Milrinone (Primacor®)

Medication	Trade name	Compatible?
Acyclovir		YES
Albumin		unknown
Ampicillin		YES
Calcium chloride		YES
Calcium gluconate		YES
Ceftriaxone	Rochephin	unknown
Chlorothiazide	Diuril	NO
Cisatracurium	Nimbex	unknown
Clindamycin		YES
Dexamethasone	Decadron	YES
Dexmedetomidine	Precedex	YES
Diazepam	Valium	unknown
DoBUTamine		YES
DoPAMine		YES
Epinephrine		YES
Famotidine	Pepcid	unknown
Fentanyl	Duragesic	YES
Fosphenytoin	Cerebyx	unknown
Furosemide	Lasix	NO
Gentamicin		YES
Heparin		YES
Imipenem/cilastatin		NO
Insulin, regular		YES
Ketamine		unknown
Magnesium sulfate		YES
Meropenem	Merrem	YES
Methylprednisolone	Solu-Medrol	YES
Metronidazole	Flagyl	YES
Midazolam	Versed	YES
Morphine		YES
Nafcillin		unknown
Norepinephrine	Levophed	YES
Phenylephrine	Neo-Synephrine	unknown
Potassium chloride		YES
Procainamide		NO
Ranitidine	Zantac	YES
Sodium bicarbonate		YES
Terbutaline		unknown
TPN + Lipids		YES
Vancomycin		YES
Vasopressin		YES
Vecuronium		YES

### Duration of study intervention and study drug discontinuation criteria:

#### In the absence of complications:

(i) Milrinone/placebo is continued until oxygenation improves and two OIs obtained at least one (up to 12) hour apart are less than 7 (or OSIs less than 3.5), then weaned according to the protocol below, or.

(ii) Milrinone/placebo is weaned and discontinued after a maximum of 3 days of therapy (total maximum duration of therapy = 72 h from the initiation of study drug).

#### Surgery and ECMO:

(i) The study drug can be continued if the patient requires ECMO.
(ii) It is recommended that the study drug be continued through surgery. If the medical service overseeing ECMO treatment or if the surgical team prefers to discontinue the study drug, this indication should be recorded and study drug weaned as per protocol.

### If serious, potentially drug-related complications occur, drug should be discontinued immediately (without weaning):

(i) The study drug is discontinued (without weaning) if the patient develops severe or life-threatening hypotension/shock. Severe hypotension is defined as a decrease in mean blood pressure > 20 mmHg compared to baseline OR receiving extensive volume therapy (>40 ml/kg during a 24 h period of the study monitoring period or escalation of vasoactive inotropic score by >20 compared to baseline).
(ii) The study drug is discontinued if the subject is detected to have a severe intracranial hemorrhage on cranial imaging (defined as unilateral or bilateral blood in the ventricles distending the ventricles or a parenchymal hemorrhage associated with a midline shift) or if patient is noted to have clinically significant extracranial bleeding.
(iii) If a significant deterioration of renal function is observed (defined as serum creatinine >2 mg/dL, increase in serum creatinine by >0.5 mg/dl compared to baseline prior to study drug, urine output <0.25 ml/kg/h over 12 h), milrinone/placebo infusion is discontinued immediately without weaning. Milrinone is excreted by the kidney and renal dysfunction can result in accumulation of milrinone.
(iv) Arrhythmias have been reported in adults during milrinone therapy. The study drug is discontinued in the presence of a heart rate rhythm abnormality including persistent bradycardia (heart rate ≤ 70/min) or tachycardia (heart rate > 220). The study drug is also discontinued if the heart rate abnormality is treated with anti-arrhythmic medications, cardioversion or defibrillation or associated with a > 5 mmHg decrease in mean blood pressure from the time prior to the onset of arrhythmia

The study drug can be discontinued (with or without weaning) by the clinical team at any time point if the team is concerned that the study drug poses a risk to the infant. An indication for discontinuation should be recorded.

#### Weaning of study drug

Once discontinuation criteria are reached, milrinone/placebo is weaned by 0.33 μg/kg/min every two hours and discontinued (total duration of wean 2 h if the patient is on 0.66 μg/kg/min and abrupt cessation of the medication infusion if the patient is on 0.33 μg/kg/min). If the subject is on 0.33 μg/kg/min, no weaning is required and the study drug is stopped at 72 h after initiation.

After the patient has met criteria for study drug discontinuation, study drug is discontinued and cannot be reinitiated even if the indication for initial discontinuation is resolved (for e.g., hypotension is resolved and blood pressure is normal). The clinical team may start open-label milrinone.

#### Open-label milrinone use

Immediately after completion of study intervention, open-label milrinone is permitted at the discretion of the clinical team.

#### Clinical criteria for ECMO

The decision to place an infant with CDH on ECMO *is entirely up to the clinical team*. Many centers prefer to use ECMO for preoperative stabilization. [42].

#### Cranial ultrasounds

An initial cranial ultrasound will be obtained prior to initiation of study drug or within 4 h of commencement of the study drug. A second cranial ultrasound will be obtained preferably within 24 h (maximum - within 96 h) of completion of the study drug. Both these cranial ultrasounds are covered by the study budget. Additional head imaging may be performed based on clinical indications (but are not covered by the study budget).

#### Echocardiography

Data from clinical echocardiograms will be recorded (maximum 4 studies – one prior to study drug initiation, two during study drug infusion and one after study drug infusion). The echocardiograms will be used to assess if there is shunting at the atrial level (and if present, the direction of the shunt), the presence of PDA (direction and velocity of shunt), position of the interventricular septum, the presence of tricuspid regurgitation (if present the pressure gradient will be recorded), chamber measurements, and any other relevant findings. These echocardiograms are obtained for clinical indications in this pilot trial.

The design of this pilot study includes randomization based on oxygenation status and not on underlying cardiac physiology. For the sake of simplicity, no echocardiograms are mandated prior to randomization. It is possible that subjects with CDH-PH without LV dysfunction and strict pulmonary arterial hypertension respond well to inhaled pulmonary vasodilator such as iNO. Subjects with LV dysfunction may have ductal dependent systemic perfusion and as such have poor outcomes with initiation of iNO. In addition, the presence of increased end diastolic pressure in the subgroup with LV dysfunction may lead to left atrial hypertension and post-capillary (or pulmonary venous) hypertension, leading to pulmonary edema aggravated by pulmonary vasodilators such as iNO. The subset of infants with LV dysfunction may respond better to milrinone and potentially deteriorate with the use of inhaled pulmonary vasodilators such as iNO. Information obtained from echocardiograms will be used to plan a future definitive trial. Echocardiographic evidence of LV dysfunction will be considered as an inclusion criterion for the definitive trial.

### Follow-up

The infants will be followed during their inpatient stay; a phone follow up will be performed at 4, 8 and 12 months to evaluate the infant’s pulmonary and nutritional status.

### Safety reporting

#### Proposed frequency of analysis, including any interim analysis

An independent data safety and monitoring committee (DSMC) has been appointed and consists of a chair and scientific and methodological experts including a neonatologist, a surgeon, a statistician, and an ethicist. The DSMC is independent of the trial investigators and sponsors. The DSMC will monitor the progress of the trial on a routine basis for futility and review the accruing safety data.

### Ethical approval

The protocol and the informed consent forms will be submitted to local institutional review boards (IRB) and ethics committees for review and approval. After initial approval, any updates to the protocol along with safety and progress reports will be submitted for review to the IRB/ethics committee at least annually and within 6 month of study termination or completion. The reports will include summaries of DSMC review of safety and efficacy.

### Survey of CDH patients in the network current vasodilator therapy

A brief survey of the NRN centers was conducted in early 2013 (there were 18 centers in the network during that cycle. Following the renewal in 2016, the number of centers was reduced to 15). The number of patients with CDH, survival, ECMO utilization and information on pulmonary vasodilator therapy was collected and is shown in Table 3. Inhaled NO is the most commonly used pulmonary vasodilator in CDH-PH and more than half of CDH patients received iNO during some part of their hospital course in the NICU. Significant center variation was observed in iNO use for CDH-PH (varying from 13 to 100% of patients). Milrinone (IV) was used in 17% of patients (range 0 to 70%) and sildenafil (IV and PO) were used in 17% of CDH-PH patients (range 0 to 73%). The primary physician managing pulmonary vasodilator therapy for CDH-PH also varied from center to center with neonatologists managing this therapy in nearly two-thirds of centers (Table 3).

**Table 3:** Infants with congenital diaphragmatic hernia in the Neonatal Research Network

	2011	2012	Average	Range among centers
Total CDH admissions	175	162	169	
CDH ≥ 36 0/7 week PMA	142	133	138 (81%)	
Inhaled NO in the preoperative period among CDH ≥ 36 0/7 weeks PMA	53	55	54 (39%)	0–100%
Other medications used in the preoperative period among CDH ≥ 36 0/7 weeks PMA				
Milrinone	17	29	23 (17%)	0–70%
Sildenafil	27	21	24 (17%)	0–73%
Flolan ® (inhaled epoprostenol)	8	9	9 (6%)	0–68% ^a^
Flolan ® (intravenous epoprostenol)	6	3	5 (3%)	0–25% ^a^
Alprostadil (intravenous PGE1)	6	5	6 (4%)	0–50%
Epinephrine	2	0	1 (1%)	0–25% ^a^
ECMO among all CDH	59	56	58 (34%)	8–87%
ECMO among CDH ≥ 36 0/7 weeks PMA	51	48	50 (36%)	9–59%
Survival to discharge				
- All CDH	111	117	114 (68%)	40–100%
- CDH ≥ 36 0/7 weeks PMA	94	101	98 (71%)	53–100%
Concern regarding use of iNO in CDH	Yes – 8; No – 11^b^			
Primary physician managing pulmonary vasodilator therapy Surgeons – 4; Neonatologists – 10; Joint (surgeons + neonatologists – 3); Pediatric Intensivists – 1				

^a^ These medications were used in one center only within the Network

^b^ One center with two hospitals had a different response at each hospital

### Variation in practice

There is significant variation in management of CDH-PH among centers participating in the network. The general guidelines for management shown in Fig. 3 are provided to all centers. These guidelines are not mandatory.

- Preductal oxygen saturation limits of 85 to 95% (unless in 21% oxygen) after immediate postnatal stabilization.
- Conventional ventilation (preferably in the volume guarantee or a similar mode) is the preferred mode of initial ventilation. If ventilation or oxygenation cannot be maintained on conventional ventilation, the infant may be switched to a high frequency ventilator (either oscillatory ventilation or jet ventilation).
- Indications for iNO: Inhaled NO may be used for hypoxemic respiratory failure with an OI of at least >15 and clinical and/or echocardiographic evidence of CDH-PH.

### Plans for a future definitive trial

This pilot trial is being conducted to collect data on safety, oxygenation and echocardiographic response to milrinone in CDH-PH and to test the feasibility of conducting a large definitive trial. This large trial will need to be a multicenter, international trial where randomization is based on oxygenation status and echocardiographic criteria with a clinically meaningful outcome such as survival without ECMO. The estimated sample size for such a trial is likely to be close to 750–800 patients with CDH-PH.

## Conclusion

Infants with CDH continue to have high mortality with high morbidity among survivors. Survival to hospital discharge among tertiary care centers with the NICHD Neonatal Research Network is 68%. Although no evidence-based guidelines are available for pulmonary vasodilator therapy, multiple vasodilators including iNO are commonly used within the network. The primary team managing pulmonary vasodilator therapy is distributed between neonatal and surgical services. Optimal management during the preoperative period focusing on gentle ventilation, avoiding oxygen toxicity, baro/volutrauma to the hypoplastic lung, cardiac support and management of pulmonary hypertension (Fig. 3) may be beneficial. Intravenous milrinone is a commonly used medication in neonatal and pediatric intensive care units and is currently used in 17% of patients with CDH within the NRN. This pilot will provide data and enable further studies evaluating pulmonary vasodilator therapy in CDH.

## Abbreviations

ASD: Atrial septal defect
cAMP: Cyclic adenosine monophosphate
CDH: Congenital diaphragmatic hernia
CDH-PH: Pulmonary hypertension associated with CDH
DSMC: Data Safety Monitoring Committee
ECMO: Extracorporeal membrane oxygenation
HRF: Hypoxemic respiratory failure
iNO: Inhaled nitric oxide
IVH: Intraventricular hemorrhage
NICHD: Eunice Kennedy Shriver National Institute of Child Health and Human Development
NICU: Neonatal intensive care unit
NRN: Neonatal research network
OI: Oxygenation index
OSI: Oxygen saturation index
PDA: Patent ductus arteriosus
PDE3: Phosphodiesterase 3
PH: Pulmonary hypertension
PMA: Postmenstrual age
PPHN: Persistent pulmonary hypertension of newborn
VSD: Ventricular septal defect
